# *SDHA* Germline Variants in Adult Patients With *SDHA*-Mutant Gastrointestinal Stromal Tumor

**DOI:** 10.3389/fonc.2021.778461

**Published:** 2022-01-04

**Authors:** Maria A. Pantaleo, Milena Urbini, Angela Schipani, Margherita Nannini, Valentina Indio, Antonio De Leo, Bruno Vincenzi, Antonella Brunello, Giovanni Grignani, Mariaelena Casagrande, Elena Fumagalli, Elena Conca, Maristella Saponara, Elisa Gruppioni, Annalisa Altimari, Dario De Biase, Giovanni Tallini, Gloria Ravegnini, Daniela Turchetti, Marco Seri, Andrea Ardizzoni, Paola Secchiero, Annalisa Astolfi

**Affiliations:** ^1^ Division of Oncology, IRCCS Azienda Ospedaliero Universitaria di Bologna, Bologna, Italy; ^2^ Department of Experimental, Diagnostic and Specialized Medicine, S.Orsola-Malpighi Hospital, University of Bologna, Bologna, Italy; ^3^ “Giorgio Prodi” Cancer Research Center, University of Bologna, Bologna, Italy; ^4^ Anatomic Pathology and Molecular Diagnostic Unit-University of Bologna Medical Center, Bologna, Italy; ^5^ Department of Medical Oncology, University Campus Bio-Medico, Rome, Italy; ^6^ Oncology 1 Unit, Department of Oncology, Istituto Oncologico Veneto IOV - IRCCS, Padova, Italy; ^7^ Division of Medical Oncology, Candiolo Cancer Institute, FPO-IRCCS, Candiolo, Italy; ^8^ Department of Oncology , University and General Hospital, Udine, Italy; ^9^ Department of Medical Oncology, Fondazione IRCCS Istituto Nazionale dei Tumori, Milan, Italy; ^10^ Department of Diagnostic Pathology and Laboratory Medicine, Fondazione IRCCS Istituto Nazionale dei Tumori, Milan, Italy; ^11^ Melano and Sarcoma Medical Treatment Unit, Istituto Europeo di Oncologia, Milan, Italy; ^12^ Department of Pathology, IRCCS Azienda Ospedaliero-Universitaria di Bologna, Bologna, Italy; ^13^ Department of Pharmacy and Biotechnology (FaBit), University of Bologna, Bologna, Italy; ^14^ Unit of Medical Genetics, IRCCS Azienda Ospedaliero Universitaria di Bologna, Bologna, Italy; ^15^ Department of Translational Medicine, University of Ferrara, Ferrara, Italy

**Keywords:** SDH-*deficient* GIST, SDHA, CT: Carney Triad, CSS: Carney-Stratakis syndrome, SDHA germinal mutations, gastrointestinal stromal tumors

## Abstract

**Background:**

SDH-*deficient* gastrointestinal stromal tumors (GIST) account for 20–40% of all KIT/PDGFRA-negative GIST and are due to mutations in one of the four *SDH*-complex subunits, with *SDHA* mutations as the most frequent. Here we sought to evaluate the presence and prevalence of *SDHA* variants in the germline lineage in a population of *SDHA*-*deficient* GIST.

**Methods:**

Germline *SDHA* status was assessed by Sanger sequencing on a series of 14 patients with gastric *SDHA*-*deficient* GIST.

**Results:**

All patients carried a germline *SDHA* pathogenic variant, ranging from truncating, missense, or splicing variants. The second hit was the loss of the wild-type allele or an additional somatic mutation. One-third of the patients were over 50 years old. GIST was the only disease presentation in all cases except one, with no personal or familial cancer history. Seven metastatic cases received a multimodal treatment integrating surgery, loco-regional and medical therapy. The mean follow-up time was of 10 years, confirming the indolent clinical course of the disease.

**Conclusion:**

*SDHA* germline variants are highly frequent in SDHA-*deficient* GIST, and the disease may occur also in older adulthood. Genetic testing and surveillance of *SDHA*-mutation carriers and relatives should be performed.

## Introduction

SDH-*deficient* gastrointestinal stromal tumors (GIST) account for 20–40% of all KIT/PDGFRA-negative GIST (1). Several evidences have suggested that SDH-*deficient* GIST exclusively arise from the stomach with mainly multifocal primary localization, frequently present lymph node involvement, generally affect younger population, and above all, have an indolent behavior even with metastatic disease (2). SDH deficiency in GIST is defined by the loss of expression of SDHB protein at immunohistochemistry and is mainly due to mutations in the four SDH mitochondrial complex subunits: *SDHA*, *SDHB*, *SDHC*, and *SDHD* (3). *SDHA* mutations are the most frequent among SDH alterations in GIST, accounting approximately for half of the cases. Other rare epigenetic events involve the recurrent aberrant DNA methylation of *SDHC* seen in GIST associated to the Carney triad (CT), which is a rare condition with synchronous or metachronous occurrence of GIST, paragangliomas, and pulmonary chondromas (4–6).

Most of *SDH* mutations in GIST are germline, in particular germline mutations in *SDHB*, *SDHC*, and *SDHD* occur in about 20–30% of SDH-*deficient* disease and may be referred to as a Carney-Stratakis syndrome (CSS) (4). This syndrome was firstly described in 2002 as a hereditary condition characterized by the occurrence of GIST and paraganglioma. Germline *SDHA* pathogenic variants have been rarely described in apparently sporadic cases (7, 8). Currently, germline testing is recommended for all SDH-*deficient* GIST, but no clear guidelines for genetic counseling and follow-up of *SDH* mutation carriers and relatives have yet been released (9). Moreover, regarding *SDHA* mutations, many issues are still unclear such as the germline status and the clinical implications that are not yet linked to well-defined hereditary syndrome. The aim of this work is to evaluate the presence and the prevalence of *SDHA* variants in the germline lineage in an SDH-*deficient* GIST population harboring *SDHA* somatic mutations.

## Materials and Methods

### Patients

Sixteen patients with a gastric *SDHA*-mutant GIST were studied. All cases were negative for SDHB immunohistochemistry, and *SDHA* mutations were assessed by Sanger sequencing of coding exons and exon-flanking regions as previously reported (10).

The tumor and patients’ characteristics are reported in **Table 1**. The study was performed in accordance with the Declaration of Helsinki protocols. The study was reviewed and approved by the local Institutional Ethical Committee of Azienda Ospedaliero-Universitaria Policlinico S. Orsola-Malpighi, Bologna, Italy (approval number 113/2008/U/Tess), and informed consent was provided by all living patients.

**Table 1:** Patients and tumor characteristics.

Pts N°	Age	Gender	Primary Site	Multifocality	Disease Status at Diagnosis	Follow-up time	Patient Status
#1	28	F	Stomach	Yes	Metastatic	15.8 yrs	AWD
#2	30	M	Stomach	No	Metastatic	13.0 yrs	AWOD
#3	31	F	Stomach	No	Localized	7.4 yrs	AWOD
#4	61	M	Stomach	No	Localized	3.3 yrs	AWOD
#5	21	F	Stomach	Yes	Localized	22.8 yrs	AWOD
#6	39	F	Stomach	No	Metastatic	14.7 yrs	AWD
#7	37	F	Stomach	No	Localized	9.6 yrs	AWOD
#8	38	M	Stomach	No	Localized	5.0 yrs	AWOD
#9	70	F	Stomach	No	Localized	10.0 yrs	AWOD
#10	66	F	Stomach	No	Localized	2.2 yrs	AWD
#11	17	M	Stomach	No	Localized	22.7 yrs	AWD
#12	55	F	Stomach	No	Metastatic	12.0 yrs	DOD
#13	50	M	Stomach	No	Localized	9.6 yrs	AWOD
#14	17	M	Stomach	No	Localized	4.8 yrs	AWOD
#15	54	F	Stomach	NA	NA	NA	NA
#16	18	F	Stomach	Yes	Localized	NA	NA

AWD, alive with disease; AWOD, alive without disease; DOD, died of disease; NA, not available.

### Sanger Sequencing

For germline analysis, DNA was extracted from peripheral blood or FFPE normal tissue with the QiaAmp mini or micro kit (Qiagen). For somatic analysis, manual macrodissection of the tumor area was performed using a scalpel on areas selected by an expert pathologist on FFPE slides. At least 70% tumor enrichment was required for sample inclusion, and DNA was extracted using QiaAmp micro kit. *SDHA* variants were identified by Sanger sequencing. Exonic and flanking intronic regions of *SDHA* were amplified with FastStart TAQ polymerase (Roche) and sequenced on both strands using the Big Dye Terminator v1.1 Cycle Sequencing kit (Applied Biosystems) on ABI 3730 Genetic Analyzer (Applied Biosystems). Primer pairs were designed with Primer Express 3.0 Software (Applied Biosystems) to specifically amplify *SDHA* exons and not the related pseudogenes (10). Germline mutational analysis was performed on peripheral blood in nine cases and on matched normal tissue extracted from FFPE in five cases. Unfortunately, in two cases the matched normal counterpart was not available. Allele frequency in the general population was reported from the Genome Aggregation Database (gnomAD) v2.1.1, reporting data from 141,456 individuals. Variant classification was performed following ACMG recommendations using the VarSome shared data resource (https://varsome.com/).

## Results

### Germline Mutational Analysis

**Figure 1:**
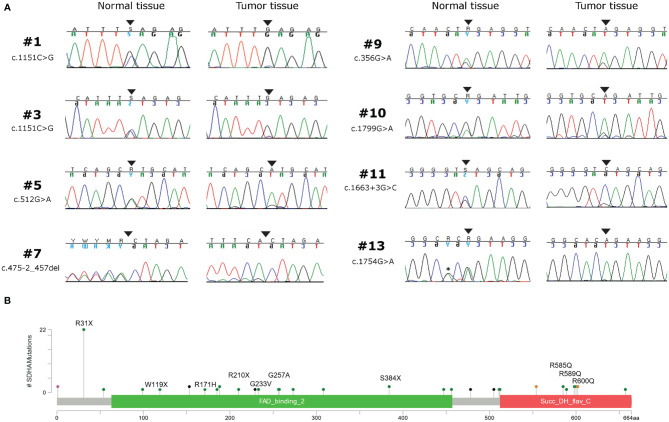
**(A)** Chromatograms showing the loss of heterozygosity of *SDHA* germline variants in tumor tissue of eight GIST patients. Black arrows indicate the position of the mutation; the asterisk indicates a silent single nucleotide polymorphism. **(B)** *SDHA* germline variant frequency of GIST patients coming from this series (written protein mutation) and from previous reports (3, 7, 8, 11–17), shown as lollipop plot.

Germline variants were identified in all 14 patients for which the normal counterpart was available (**Table 2**). Five cases harbored truncating non-sense variants (Ser384*, Arg31*, Trp119*, Arg210*), seven other cases carried missense variants (Gly233Val, Arg171His, Arg589Gln, Gly257Ala, Arg600Gln, Arg585Gln), and two harbored exon-flanking intronic variants predicted to affect splicing. In particular, the prediction with the tool Alternative Splice Site Predictor (ASSP) revealed that the c.457-2_457del is likely to generate an alternative acceptor splice site of exon 5, while the c.1663+3 G>C probably leads to the loss of donor splice site at exon 12 with consequent intron retention. All the two splice site alterations, as predicted, lead to a frameshift with a stop codon in the corresponding protein sequence. All the truncating variants, one splice-site, and two missense variants were classified as “Pathogenic” or “Likely Pathogenic” by the ACMG recommendations (**Table 2**). Conversely, the other five variants were classified as “Uncertain Significance” but were all predicted as damaging by the computational prediction algorithms implemented in Varsome. In eight cases, tumor DNA showed the loss of the corresponding wild-type allele, thus displaying homozygosity for the germline variant, while in the other six cases compound heterozygosity for an additional somatic mutation was detected (Arg589Trp, Arg451Cys, Arg171Cys, Arg585Gln, Thr308Met, and Gln176*) (**Figure 1A**). In both patients for which normal DNA was not available, tumors carried two mutational hits on *SDHA*. Germline *SDHA* variants identified in this study, along with those identified in previous reports on GIST patients, are summarized in **Figure 1B**. As expected, mutations are scattered along the whole coding sequence, with a peak frequency of the Arg31* variant, recurrently identified in previous studies (3, 7, 11–18) and present in one case in our series.

**Table 2:** Mutational analysis of germline and tumors in SDHA-*deficient* GIST patients.

Pts N°	Normal Counterpart Germline Variant						Tumor Tissue Somatic Mutation			
	Sample	Mutation	Variant Classification	Exon	Status	GnomAD Frequency	Sample	Mutation	Variant Classification	Exon
#1	PB	c.1151C>G; p.Ser384*	P	9	Hetero	7.96x10^-6^	FF	LOH	P	9
#2	PB	c.91C>T; p.Arg31*	P	2	Hetero	2.09x10^-4^	FF	c.1765C>T; p.Arg589Trp	LP	13
#3	PB	c.1151C>G; p.Ser384*	P	9	Hetero	7.96x10^-6^	FF/FFPE	LOH	P	9
#4	PB	c.698G>T; p.Gly233Val	UNC	6	Hetero	ND	FFPE	c.1351C>T; p.Arg451Cys	UNC	10
#5	PB	c.512G>A; p.Arg171His	UNC	5	Hetero	1.41x10^-5^	FFPE	LOH	UNC	5
#6	PB	c.1766G>A; p.ArgR589Gln	LP	13	Hetero	ND	FF	c.511C>T; p.Arg171Cys	UNC	5
#7	PB	c.457-2_457del	P	5	Hetero	ND	FFPE	LOH	P	5
#8	PB	c.770G>C; p.Gly257Ala	P	6	Hetero	ND	FFPE	c.1754G>A; p.Arg585Gln	UNC	13
#9	FFPE	c.356G>A; p.Trp119*	P	4	Hetero	ND	FF	LOH	P	4
#10	FFPE	c.1799G>A; p.Arg600Gln	UNC	14	Hetero	2.09x10^-5^	FFPE	LOH	UNC	14
#11	PB	c.1663+3G>C	UNC	12	Hetero	1.59x10^-5^	FFPE	LOH	UNC	12
#12	FFPE	c.1799G>A; p.Arg600Gln	UNC	14	Hetero	2.09x10^-5^	FFPE	c.923C>T; p.Thr308Met	UNC	8
#13	FFPE	c.1754G>A; p.Arg585Gln	UNC	13	Hetero	7.97x10^-6^	FFPE	LOH	UNC	13
#14	FFPE	c.628C>T; p.Arg210*	P	6	Hetero	3.98x10^-6^	FFPE	c.526C>T; p.Gln176*	P	5
#15	NA	NA	NA	NA	NA	NA	FFPE	c.923C>T; p.Thr308Met + c.1741G>A; p.Gly581Arg	UNC + LP	8+13
#16	NA	NA	NA	NA	NA	NA	FFPE	c.1255G>A; p.Gly419Arg + c.1690G>A; p.Glu564Lys	UNC + UNC	9+13

The cDNA and protein mutation in the normal germline and the second somatic mutation in GIST is reported, along with the variant classification following ACMG recommendations, the allelic status, and the allelic frequency in the general population (gnomAD database). PB, peripheral blood; FF, fresh-frozen; NA, not available; ND, not detected; P, pathogenic; LP, likely pathogenic; UNC, uncertain significance.

### Clinical Correlation

**Figure 2:**
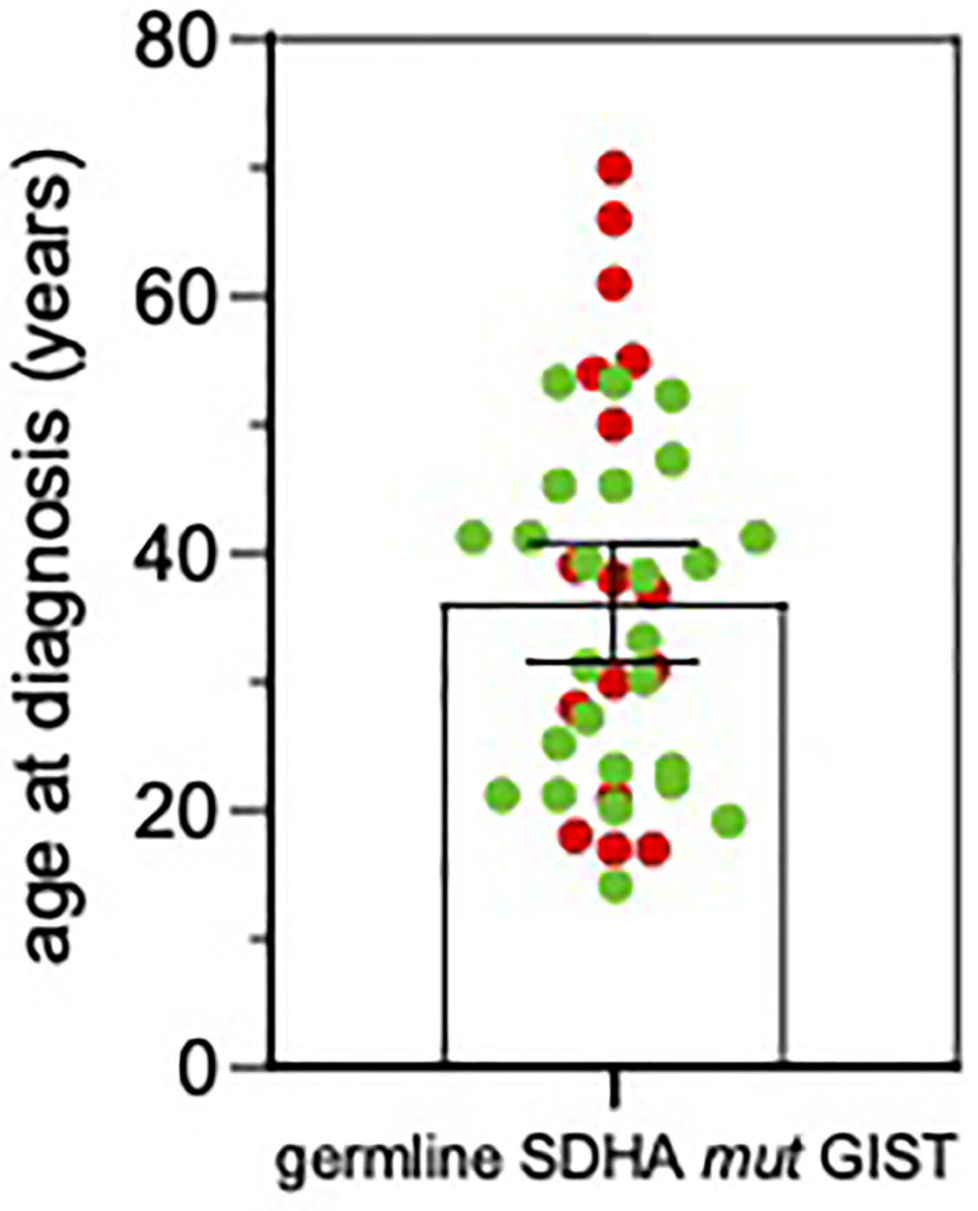
Age at GIST onset in *SDHA*-variant carriers. The mean and SEM are shown. Red, patients from this series; Green, patients from previously published reports (3, 7, 11–18).

Average age at diagnosis was 39.5 ± 4.5 years (range 17–70). Ten cases were young-adults (range: 17–39 years old) and 6 patients were older adults (>50 years old; range: 50–70). Combining our series with the previously published reports (3, 7, 8, 11–18), it emerges that GIST can arise at very different age ranges in *SDHA*-variant carriers, since mean age at diagnosis is 36.0 ± 2.3 years, but up to 22% of patients are being diagnosed at more than 50 years old (**Figure 2**). In all cases of adult patients, the GIST was unifocal. In the whole series, four patients displayed metastases already at the time of diagnosis, while all other harbored localized disease. In all cases except one, the GIST was the only disease presentation, and no personal or familial cancer history was revealed. Only one case of 61 years of age at diagnosis reported having been affected by paraganglioma in the past (more than 10 years before), but unfortunately the biological material was not available for a pathologic revision and genetic testing. Except for two cases lost at follow-up, the mean follow-up time was 10.9 years, ranging between 2.2 to 22.8 years. Among localized cases, three developed a recurrence with multiple metastases. Altogether, seven metastatic cases received a high-complexity treatment with standard medical therapy integrated with surgery and/or other loco-regional therapy (radiofrequency of liver metastasis and also chemoembolization in one case). Among these seven metastatic cases, one died of disease and one is currently free of disease through complete surgical removal of liver metastases.

## Discussion

In a population of SDH-*deficient* GIST harboring *SDHA* somatic mutations, we found germline *SDHA* variants in all cases for which normal DNA was available, and these findings underline that germline mutations in *SDHA* are highly frequent in SDHA-*deficient* GIST. Besides the non-sense and missense mutations, we also found two cases harboring damaging splice-site mutations (c.457-2_457 and c.1663+3 G>C) leading to a predicted premature protein sequence truncation. Overall, in our series we found seven truncating germline variants (five non-sense and two splice-site) present in all but one case in young patients. Conversely, four out of seven germline missense variants, whose effect on protein function and stability is harder to be assessed, were identified in older patients (more than 50 years old at diagnosis). Moreover, we did not find a high frequency of the Arg31* variant, whose recurrent identification in previous studies even led to the assumption of a putative founder effect (3, 7, 14, 16–18). Apart from the recurrent Arg31*, the high diversity of the variant type and position seen in our and in previous series supports a view in which *SDHA* can be inactivated by mutations scattered throughout its whole genomic sequence, suggesting that *SDHA* genetic analysis must be performed entirely and by qualified high-volume molecular diagnostic centers, as already suggested in other settings (19).

Lastly, all the described patients carried two mutational events at the *SDHA* locus, either the loss of the wild-type allele or a second somatic event in compound heterozygosity, in full agreement with the two-hit hypothesis of tumor suppressor genes inactivation. Therefore, this suggests the possibility of a germline first mutation also in the two cases of our series for which the matched normal DNA counterpart was not available. Interestingly, an in-depth biochemical study of the functional effects of *SDHA* variants of unknown significance supported the pathogenicity of two somatic mutations (p.Arg451Cys e p.Gly419Arg) classified as uncertain by ACMG recommendations, while it did not support the loss of function of the Arg171His variant (20).

Furthermore, our study definitely confirms the highly relevant association between germline *SDHA* pathogenic variants and GIST onset, which is supported by many previous studies reporting the development of GIST as the only cancer disease in *SDHA* germline-variant carrier population (3, 7, 13, 14, 18). In our series, only one case reported having been affected by paraganglioma more than 10 years before, but the diagnosis was not now revised. Recently, a large case-control study has estimated the penetrance of SDHx variants for PPGL, indicating a 1.7% lifetime disease penetrance for SDHA pathogenic variants (21). Up to now, the onset of other tumor types in the context of *SDHA*-mutant GIST appears to be very rare and limited to the concurrent description of paraganglioma in one case, of pulmonary chondroma in two cases, and of the full Carney triad in one patient (8, 14). Indeed, it was shown that mutations in *SDHA* account for around 30% of all KIT/PDGFRA-WT GIST (10), while being responsible for <1% of all pheochromocytoma and paraganglioma cases (PPGL), as opposed for example to *SDHB* that accounts for about 10% of all PPGL (22, 23). The only study that up to now suggesting the possible role of *SDHA* as a predisposing factor for other tumor types reported the development of neuroblastoma in one *SDHA*-mutation carrier showing the inactivation of the second allele (17). Therefore, our result, coupled with previous data and with the identification of a higher-than-expected frequency of *SDHA* germline pathogenic variants in a cohort of cancer patients with respect to healthy individuals from the Exome Aggregation Consortium (ExAC) database (17), further endorses the view of *SDHA* as a cancer gene mainly predisposing to GIST development. This aggregate result underscores the different tumor spectrum of *SDHA*-germline variant carriers and Carney Stratakis Syndrome patients, which are known to harbor *SDHB*, *SDHC*, or *SDHD* mutations, and to develop invariably the association of GIST and paraganglioma during their life course (24).

Actually, in literature and in our experience, a clear syndrome has not been clearly defined since all these cases appear to be sporadic without other neoplasms except from GIST, and therefore it still remains unclear if *SDHA* mutations should be accounted in the CSS. CSS by definition is due to *SDHB*, *SDHC*, and *SDHD* mutations and is characterized by the combination of GIST and paraganglioma inherited in an apparently autosomal dominant manner and with incomplete penetrance. Paragangliomas were described to be multicentric and GIST multifocal. Both these clinical presentations support the inherited nature of this tumor predisposition (4). Currently, germline testing is recommended for all SDH*-deficient* GIST including *SDHA* in some clinical practice guidelines (9, 25), but no clear protocol for genetic counseling and follow-up of *SDHx* variant carriers and relatives has been released, especially for those carrying a germline *SDHA* pathogenic variant not yet linked to well-defined hereditary syndrome.

Regarding the clinical findings in GIST patients with *SDHA* germline mutation, our series confirms the stomach as unique site of GIST onset but underlines a higher heterogeneity for the multifocality development and also for the metastatic presentation at diagnosis. Larger series and international efforts should be required to better define a phenotype/genotype correlation in this subset of disease between GIST features, other tumor development, and *SDH* genotyping in tumor/germline. No conclusive consideration can be made regarding the survival rates because of the heterogeneity of clinical presentations and the multiple treatments received in cases with metastatic disease. After all, our series confirms the indolent clinical course and long survival expectations since half of population presents a follow-up higher than 10 years, and four of these patients present a metastatic disease.

This study therefore shows that, differently from previously stated, the *SDHA*-mutant GIST patient is almost exclusively a germline *SDHA*-variant carrier that is prone to develop the tumor throughout his entire life and not just in his early adulthood (7, 10–12, 14). In fact, 38% of the patients from our series were older than 50 years, with peaks up to 70 years old. Actually, this result is supported by previous studies that occasionally reported age ranges up to 70 years old and subsets of *SDHA*-mutant GIST patients of more than 50 years old (3, 7, 13, 14, 16). This fact has very important implications supporting the need for genetic counseling, since even though complete pedigree analysis of *SDHA* mutation carrier families is still lacking and the clinical insights suggest a low penetrance of *SDHA* mutation in affected families (12, 26), the notion of a lifelong risk for GIST development urges the need for genetic testing and permanent clinical surveillance of *SDHA*-variant carriers.

As the process of referral to genetic counseling of the patients included in the study is still ongoing, conclusive information on documented history of cancer in relatives, segregation of the variant in the family, and clinical assessment of carriers, who will be included in management programs according to updated recommendations, are not yet available. Now, this may represent a formal limitation of the study, however not modifying its substantial conclusions.

In conclusion, germline pathogenic variants in *SDHA* are highly frequent in SDHA-*deficient* GIST in young and adult patients, and the disease may occur also in older adulthood. Genetic counseling of *SDHA-*variant carriers and relatives should be planned, and their clinical follow-up should be accurately defined.

## Data Availability Statement

The datasets presented in this study was submitted to ClinVar (SUB10460221). Variants identified are available under accession numbers from SCV002026125 to SCV002026144.

## Ethics Statement

The studies involving human participants were reviewed and approved by Ethical Committe of Pol. Sant’Orsola Malpighi, University of Bologna. The patients/participants provided their written informed consent to participate in this study.

## Author Contributions

Conceptualization: MP, MU, AAs, and VI. Data curation: VI, AAs, MN, and MSa. Formal Analysis; MU, VI, AS, AAs, and GR. Funding acquisition: MP and MN. Investigation: MU, VI, AS, AAs, BV, AB, GG, MC, EF, EC, MSa, AL, and GT. Methodology: MU, VI, AS, AAs, EG, AAl, and GR. Project administration: MP. Software: VI, GR, and MU. Supervision: MP, AAs, AAr, and PS. Validation: MU, VI, AS, AA, GR, AL, EG, AAl, and DB. Visualization: DT, MSe, and GT. Writing—original draft: MP, AAs, and VI. Writing—review and editing: MP, AAs, MU, and VI. All authors contributed to the article and approved the submitted version.

## Funding

This work was supported by Fondazione Carisbo, Bologna (Bando Ricerca medica traslazionale e clinica 2019), Italy, and by a research donation in memory of Alberto Arenghi, Caravaggio, BG, Italy. The funders have no role in the design of the study, the collection, analysis, and interpretation of data, the writing of the manuscript, or submission of the manuscript for publication.

## Conflict of Interest

The authors declare that the research was conducted in the absence of any commercial or financial relationships that could be construed as a potential conflict of interest.

## Publisher’s Note

All claims expressed in this article are solely those of the authors and do not necessarily represent those of their affiliated organizations, or those of the publisher, the editors and the reviewers. Any product that may be evaluated in this article, or claim that may be made by its manufacturer, is not guaranteed or endorsed by the publisher.

## Abbreviations

GIST, gastrointestinal stromal tumor; CT, Carney triad; CSS, Carney-Stratakis syndrome; gnomAD, Genome Aggregation Database; PPGL, pheochromocytoma and paraganglioma; ExAC, Exome Aggregation Consortium.

## References

[1] Janeway KA Kim SY Lodish M Nosé V Rustin P Gaal J. Defects in Succinate Dehydrogenase in Gastrointestinal Stromal Tumors Lacking KIT and PDGFRA Mutations. Proc Natl Acad Sci USA (2011) 108(1):314–18. doi: 10.1073/pnas.1009199108 PMC3017134 21173220

[2] Pantaleo MA Lolli C Nannini M Astolfi A Indio V Saponara M. Good Survival Outcome of Metastatic SDH-Deficient Gastrointestinal Stromal Tumors Harboring SDHA Mutations. Genet Med (2015) 17(5):391–5. doi: 10.1038/gim.2014.115 25188872

[3] Wagner AJ Remillard SP Zhang Y-X Doyle LA George S Hornick JL. Loss of Expression of SDHA Predicts SDHA Mutations in Gastrointestinal Stromal Tumors. Mod Pathol (2013) 26(2):289–94. doi: 10.1038/modpathol.2012.153 22955521

[4] Stratakis CA Carney JA. The Triad of Parangangliomas, Gastric Stromal Tumors and Pulmonary Chondromas (Carney Triad), and the Dyad of Parangangliomas and Gastric Stromal Sarcomas (Carney-Stratakis Syndrome): A Molecular Genetics and Clinical Implications. J Int Med (2009) 266(1):43–52. doi: 10.1111/j.1365-2796.2009.02110.x PMC3129547 19522824

[5] Killian JK Miettinen M Walker RL Wang Y Zhu YJ Waterfall JJ. Recurrent Epimutation of SDHC in Gastrointestinal Stromal Tumors. Sci Transl Med (2014) 6(268):268ra177. doi: 10.1126/scitranslmed.3009961 PMC7670881 25540324

[6] Haller F Moskalev EA Faucz FR Barthelmeß S Wiemann S Bieg M. Aberrant DNA Hypermethylation of SDHC: A Novel Mechanism of Tumor Development in Carney Triad. Endocr Relat Cancer (2014) 21(4):567–77. doi: 10.1530/ERC-14-0254 PMC4722532 24859990

[7] Miettinen M Killian JK Wang ZF Lasota J Lau C Jones L. Immunohistochemical Loss of Succinate Dehydrogenase Subunit A (SDHA) in Gastrointestinal Stromal Tumors (GISTs) Signals SDHA Germline Mutation. Am J Surg Pathol (2013) 37(2):234–40. doi: 10.1097/PAS.0b013e3182671178 PMC3545041 23282968

[8] Carrera S Beristain E Sancho A Iruarrizaga E Rivero P Mañe JM. Germline C.1A>C Heterozygous Pathogenic Variant in SDHA Reported for the First Time in a Young Adult With a Gastric Gastrointestinal Stromal Tumour (GIST): A Case Report. Hered Cancer Clin Pract (2019) 17:23. doi: 10.1186/s13053-019-0124-6 31413764 PMC6688230

[9] Casali PG Abecassis N Aro HT Bauer S Biagini R Bielack S. ESMO Guidelines Committee and EURACAN. Gastrointestinal Stromal Tumours: ESMO-EURACAN Clinical Practice Guidelines for Diagnosis, Treatment and Follow-Up. Ann Oncol (2018) 29(Suppl 4):iv68–78. doi: 10.1093/annonc/mdy095 29846513

[10] Pantaleo MA Astolfi A Urbini M Nannini M Paterini P Indio V. Analysis of All Subunits, SDHA, SDHB, SDHC, SDHD, of the Succinate Dehydrogenase Complex in KIT/PDGFRA Wild-Type GIST. Eur J Hum Genet (2014) 22(1):32–9. GIST Study Group. doi: 10.1038/ejhg.2013.80 PMC3865408 23612575

[11] Italiano A Chen CL Sung YS Singer S DeMatteo RP LaQuaglia MP. SDHA Loss of Function Mutations in a Subset of Young Adult Wild-Type Gastrointestinal Stromal Tumors. BMC Cancer (2012) 12:408. doi: 10.1186/1471-2407-12-408 22974104 PMC3503624

[12] Dwight T Benn DE Clarkson A Vilain R Lipton L Robinson BJ. Loss of SDHA Expression Identifies SDHA Mutations in Succinate Dehydrogenase-Deficient Gastrointestinal Stromal Tumors. Am J Surg Pathol (2013) 37(2):226–33. doi: 10.1097/PAS.0b013e3182671155 23060355

[13] Belinsky MG Rink L Flieder DB Jahromi MS Schiffman JD Godwin AK. Overexpression of Insulin-Like Growth Factor 1 Receptor and Frequent Mutational Inactivation of SDHA in Wild-Type SDHB-Negative Gastrointestinal Stromal Tumors. Genes Chromosomes Cancer (2013) 52(2):214–24. doi: 10.1002/gcc.22023 PMC3564228 23109135

[14] Oudijk L Gaal J Korpershoek E Van Nederveen FH Kelly L Schiavon G. SDHA Mutations in Adult and Pediatric Wild-Type Gastrointestinal Stromal Tumors. Mod Pathol (2013) 26(3):456–63. doi: 10.1038/modpathol.2012.186 23174939

[15] Jiang Q Zhang Y Zhou YH Hou YY Wang JY Li JL. A Novel Germline Mutation in SDHA Identified in a Rare Case of Gastrointestinal Stromal Tumor Complicated With Renal Cell Carcinoma. Int J Clin Exp Pathol (2015) 8(10):12188–97. PMC4680348 26722403

[16] Boikos SA Pappo AS Killian JK LaQuaglia MP Weldon CB George S. Molecular Subtypes of KIT/PDGFRA Wild-Type Gastrointestinal Stromal Tumors: A Report From the National Institutes of Health Gastrointestinal Stromal Tumor Clinic. JAMA Oncol (2016) 2(7):922–8. doi: 10.1001/jamaoncol.2016.0256 PMC5472100 27011036

[17] Dubard Gault M Mandelker D DeLair D Stewart CR Kemel Y Sheehan MR. Germline SDHA Mutations in Children and Adults With Cancer. Cold Spring Harb Mol Case Stud (2018) 4(4):a002584. doi: 10.1101/mcs.a002584 30068732 PMC6071569

[18] Pantaleo MA Astolfi A Indio V Moore R Thiessen N Heinrich MC. SDHA Loss-Of-Function Mutations in KIT-PDGFRA Wild-Type Gastrointestinal Stromal Tumors Identified by Massively Parallel Sequencing. J Natl Cancer Inst (2011) 103(12):983–7. doi: 10.1093/jnci/djr130 21505157

[19] Astolfi A Indio V Nannini M Saponara M Schipani A De Leo A. Targeted Deep Sequencing Uncovers Cryptic KIT Mutations in KIT/PDGFRA/SDH/RAS-P Wild-Type GIST. Front Oncol (2020) 10:504. doi: 10.3389/fonc.2020.00504 32391261 PMC7188756

[20] Bannon AE Kent J Forquer I Town A Klug LR McCann K. Biochemical, Molecular, and Clinical Characterization of Succinate Dehydrogenase Subunit A Variants of Unknown Significance. Clin Cancer Res (2017) 23(21):6733–43. doi: 10.1158/1078-0432.CCR-17-1397 PMC6011831 28724664

[21] Benn DE Zhu Y Andrews KA Wilding M Duncan EL Dwight T. Bayesian Approach to Determining Penetrance of Pathogenic SDH Variants. J Med Genet (2018) 55(11):729–34. doi: 10.1136/jmedgenet-2018-105427 PMC6252366 30201732

[22] Tufton N Sahdev A Drake WM Akker SA. Can Subunit-Specific Phenotypes Guide Surveillance Imaging Decisions in Asymptomatic SDH Mutation Carriers? Clin Endocrinol (Oxf) (2019) 90(1):31–46. doi: 10.1111/cen.13877 30303539

[23] Evenepoel L Papathomas T Krol N Korpershoek E De Krijger RR Persu A. Toward an Improved Definition of the Genetic and Tumor Spectrum Associated With SDH Germ-Line Mutations. Genet Med (2015) 17(8):610–20. doi: 10.1038/gim.2014.162 25394176

[24] Pasini B McWhinney SR Bei T Matyakhina L Stergiopoulos S Muchow M. Clinical and Molecular Genetics of Patients With the Carney-Stratakis Syndrome and Germline Mutations of the Genes Coding for the Succinate Dehydrogenase Subunits SDHB, SDHC, and SDHD. Eur J Hum Genet (2008) 16(1):79–88. doi: 10.1038/sj.ejhg.5201904 17667967

[25] MacFarlane J Seong KC Bisambar C Madhu B Allinson K Marker A. A Review of the Tumour Spectrum of Germline Succinate Dehydrogenase Gene Mutations: Beyond Phaeochromocytoma and Paraganglioma. Clin Endocrinol (Oxf) (2020) 93(5):528–38. doi: 10.1111/cen.14289 32686200

[26] Gill AJ. Succinate Dehydrogenase (SDH)-Deficient Neoplasia. Histopathol (2018) 72(1):106–16. doi: 10.1111/his.13277 29239034

